# Home-Based Exergaming on Preschoolers’ Energy Expenditure, Cardiovascular Fitness, Body Mass Index and Cognitive Flexibility: A Randomized Controlled Trial

**DOI:** 10.3390/jcm8101745

**Published:** 2019-10-21

**Authors:** Zan Gao, Jung Eun Lee, Nan Zeng, Zachary C. Pope, Ying Zhang, Xianxiong Li

**Affiliations:** 1 School of Kinesiology, University of Minnesota-Twin Cities, Minneapolis, MN 55455, USA; 2 Department of Applied Human Sciences, University of Minnesota, Duluth, MN 55812, USA; junelee@d.umn.edu; 3 Department of Food Science and Human Nutrition, Colorado State University, 1571 Campus Delivery, 110 Gifford Building, Fort Collins, CO 80523, USA; nanzeng@colostate.edu; 4 School of Public Health, University of Minnesota-Twin Cities, Minneapolis, MN 55455, USA; popex157@umn.edu; 5 Hangzhou Kindergarten Teacher College, Zhejiang Normal University, Hangzhou 310012, China; yingzh@zjnu.cn; 6 School of Physical Education, Hunan Normal University, Changsha 410081, China; lixianxiong@hunnu.edu.cn

**Keywords:** active video games, cognitive functions, early childhood, pediatric obesity, physical activity, health promotion

## Abstract

Purpose: The effects of exergaming-based physical activity (PA) interventions on preschoolers’ health outcomes and cognition remain largely unexplored. Therefore, we conducted a randomized controlled trial to discern the effectiveness of a home-based educational exergaming intervention on preschoolers’ energy expenditure, fitness, body mass index, and cognition. Methods: Participants were 32 preschoolers (16 girls; 59.4% Asian; M_age_ = 4.72, SD = ±0.73) recruited from the Twin Cities area in Minnesota. During baseline testing, we measured preschooler’s daily energy expenditure (EE), cardiovascular fitness, body mass index, and cognitive flexibility using validated instruments. Participants were then randomly assigned to one of two conditions: (1) the exergaming intervention condition; or (2) control condition. The intervention program requested children participate in home-based educational exergaming using the LeapTV gaming console for at least 30 min/session 5 times/week. The control condition asked children to maintain regular PA patterns without any exergaming gameplay. Each condition lasted 12 weeks. We conducted identical outcome assessments for all children at baseline and post-intervention. Results: Analysis of covariance with repeated measures yielded significant time x group interaction effects for cognitive flexibility, Wilks’ Lambda = 0.81, *F*(1, 29) = 6.98, *p* = 0.01, *η*^2^ = 0.19, suggesting children in the exergaming group demonstrated significantly greater increases in cognitive flexibility as compared to those in the control group over time. There were no significant differences for time x group changes between the two groups for EE, Wilks’ Lambda = 0.92, *F*(1, 29) = 2.29, *p* = 0.14, *η*^2^ = 0.08; cardiovascular fitness, Wilks’ Lambda = 0.96, *F*(1, 29) = 1.07, *p* = 0.31, *η*^2^ = 0.04; and BMI, Wilks’ Lambda = 0.99, *F*(1, 29) = 0.05, *p* = 0.82, *η*^2^ = 0.01. However, our data did suggest a marginal effect of time for EE, Wilks’ Lambda = 0.89, *F*(1, 29) = 3.26, *p* = 0.08, *η*^2^ = 0.08, indicating that children’s daily EE increased from baseline to post-intervention. Conclusions: Home-based educational exergaming may positively impact cognitive flexibility in preschoolers. Studies with larger sample sizes in multiple geographic locations are needed, with our study suggesting a longer intervention period might also be warranted.

## 1. Introduction

The increased prevalence of childhood obesity in the U.S. is partially due to low physical activity (PA) [1] and has significant negative consequences. Obesity and low cardiovascular fitness in children may increase risk of hypertension and hypercholesterolemia during childhood and contribute to chronic disease development in adulthood (e.g., heart disease, diabetes) [2,3]. Research has suggested that preschoolers are not physically active at the recommended daily 180 min. of PA participation [4]. This problem is particularly critical in underserved urban preschool children who demonstrate higher sedentary time and live in social contexts with limited access to PA-conducive environments such as playgrounds and sports complexes [5,6,7]. PA participation plays a key role in preventing and reducing obesity among young children [8,9,10]. The preschool years (ages 3–5 years) have been identified as a crucial time to promote healthy lifestyle habits and prevent obesity development [7]. Early childhood PA interventions may help preschoolers establish healthy lifestyle habits and thereby contribute to chronic disease prevention later in adulthood [11,12]. Limited studies, however, have focused on the effects of innovative PA interventions on urban preschoolers’ cardiovascular health and body composition.

Physical inactivity is a major risk factor for increased body composition [13]. Empirical studies have consistently shown a graded and negative relationship between body composition and PA among children and adults [14,15]. Research has also regularly noted the reciprocal effects between PA, energy expenditure (EE), and health-related physical fitness [16,17,18]. Specifically, regular participation in different types of physical activities (e.g., leisure activities and sports) has been identified as an important contributor to EE and cardiovascular fitness components including cardiovascular fitness and body composition [18,19,20]. Importantly, fit children tend to be more physically active [21]. Recent studies have also suggested that higher cardiovascular fitness and PA influence children’s cognitive functions, including executive functions (e.g., working memory, attention, cognitive flexibility) [22,23,24] and academic achievement [25]. In response, we implemented a novel PA intervention modality—educational exergaming—to improve PA (and, thus, EE), fitness, body mass index, and cognition in preschool children.

Exergaming (a.k.a., active video games) refers to movement-based video games that are also a form of exercise [26]. Despite the negative impact of sedentary video games on PA levels and fitness, exergaming has the potential to promote PA participation and improve fitness levels in children [27,28,29,30]. Over the past two decades, the fast growth of exergaming has led to the development of new interactive exercise strategies that have influenced the quality of population-based PA interventions [31,32,33,34,35,36,37]. Exergaming capitalizes on children’s interest in computer and video interaction by combining interactive gaming equipment and activities to encourage movement and PA in children [38,39,40]. For example, LeapTV exergames, a type of educational active video gaming, encourage young children to learn through motion as they jump, dance, and hop, etc. LeapTV games require whole body movement while teaching core cognitive skills for reading, mathematics, science, and problem solving, which serves as an important bridge to promote children’s cognitive development and improve fitness [41].

Researchers have investigated exergaming as a means for increasing PA and promoting a healthy lifestyle in a variety of populations [42,43,44,45,46,47]. Among these studies, home-based exergaming interventions have demonstrated the most promise in promoting children’s PA levels [48,49]. However, previous home-based exergaming studies targeted only older children and adolescents (age range: 8–14 years), missing an opportunity to apply exergaming interventions in early childhood to improve health. Indeed, despite the growing market of developmentally appropriate exergames for preschoolers, a paucity of literature has examined the effects of exergaming on preschoolers’ health outcomes [50]. It is also noteworthy that most prior studies focused primarily on physiological or psychological outcomes exclusively and have also not incorporated any assessment of cognitive functions [28,36]. Thus, a need exists to examine the concurrent effects of exergaming on other important aspects of child development (e.g., EE, cardiovascular fitness, body mass index, and cognitive flexibility). In addition, it is apparent that exergaming promotes PA through active screen time which consequently intrigues the different effects of screen time (active vs. passive) on preschool children. It is recommended that preschoolers should spend a maximum of 2-h in screen entertainment on a daily. Yet, preschoolers have been reported to spend greater than 2 h engaged in TV/video viewing per day [51], with up to 75% of preschooler’s waking comprised of sedentary activities [52]. For example, U.S. 4–5 year olds spent 90 min per day in passive screen time (TV, video, DVD, computers) with an additional 90 min in other sedentary activities (reading, drawing, and music) [4]. Therefore, in the present study we encouraged preschool children in the intervention group to replace passive screen time with active screen time (i.e., educational exergaming).

This study’s purpose was to examine the effects of a home-based educational exergaming intervention on urban preschool children’s EE, cardiovascular fitness, body mass index, and cognitive flexibility compared to the usual daily practice (control) in a randomized controlled trial. Study observations may improve our understanding of how innovative technology-based PA approaches could be utilized at home to promote health and optimal development among preschoolers.

## 2. Methods

### 2.1. Research Design

**Figure 1:**
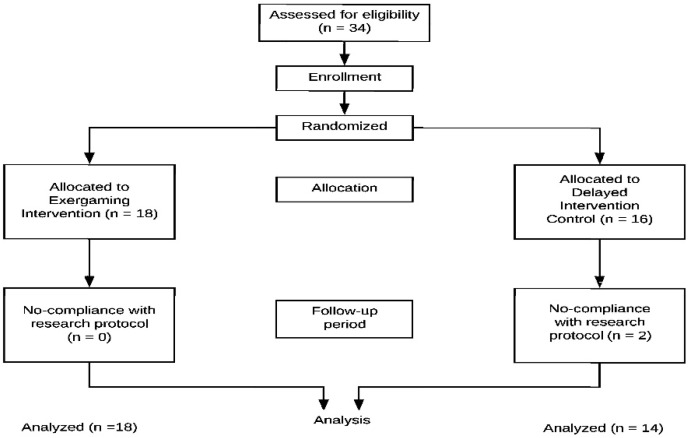
Chart of the trial.

We used a 12-week randomized controlled two-arm trial design in which each child was assigned to one of two conditions at baseline: (1) intervention condition; or (2) control condition. The intervention program requested children participate in home-based educational exergaming using the LeapTV gaming console for at least 30 min/session 5 times/week beyond their usual PA. The control condition asked children to maintain regular PA patterns without any exergaming gameplay, with parents advised to not change their children’s regular PA routine during their child(ren)’s time in this condition. Each condition lasted 12 weeks (See Figure 1). We monitored intervention fidelity via periodic home visits. All children underwent identical assessments in our lab at baseline and post-intervention.

### 2.2. Participants

Sample size was calculated using G*Power 3.1 (http://www.gpower.hhu.de/en.html) [53]. It was suggested that 32 participants would be sufficient for 80% power (α = 0.05, effect size = 0.05) to test the primary outcome (i.e., EE). We recruited children via university- and community-based fliers, university email lists, and word of mouth, and we monitored how many parents and children comprised each family using a spreadsheet. Inclusion criteria for the families were: (1) child(ren) aged 4–6 years old; (2) English-speaking; (3) a home TV with HDMI port or a TV with Video Graphics Array (VGA) port and VGA to HDMI converter adapter; (4) child(ren) possessing no mental or physical disabilities; (5) parental interest in study participation; (6) lived within 20 miles of the University; (7) willing to complete the home-based educational exergaming intervention for 12 weeks; and (8) not moving out of the area within the next 6 months. Following screening, a total of 32 preschool children from 22 families in a Midwestern metropolitan city were enrolled in this study.

### 2.3. Outcome Measures

*Demographic and Anthropometric Data*. Parents completed a demographic information sheet that gathered information on their child(ren)’s age, gender, and race in addition to the parent’s education level (e.g., high school, college, graduate, etc.) and annual income. We measured children’s height to the nearest 0.5cm using a Seca stadiometer (Seca, Chino, CA, USA) and assessed weight with a Tanita BC-558 IRONMAN^®^ digital weight scale (Detecto, Web City, MO, USA) to the nearest 0.5 kg. Body mass index was calculated from measured height and weight.

*Energy Expenditure*. ActiGraph GT3X+ accelerometers (Pensacola, FL, USA) estimated children’s daily EE. The ActiGraph has been reported as a valid and reliable measure of PA and EE among children in free-living settings [54,55]. Children wore the accelerometers for 5 full days (3 weekdays, 2 weekend days) [54] on their right hip, attached by an elastic belt, during waking hours, except for time spent bathing and engaging in other activities involving water. Acceptable inclusion criteria for the PA data were the recording of an average of 10 h of accelerometer data per day over the 5-day assessment period [54,55,56]. We evaluated accelerometer wear compliance using Trost recommendations [55]. In this study, we set activity counts at 5-s epochs, with activity counts categorized in durations of sedentary, light, moderate, and vigorous PA using empirically-based cut points that define different intensities of preschool children’s PA [57]. Participants’ kilocalories per day was used as their daily EE. An incentive gift card ($25) was given to each family for successful return of the accelerometers and download of completed data from the ActiGraph accelerometers after each testing cycle.

*Cardiovascular Fitness*. Children’s cardiovascular fitness was measured using the 3-Minute Step Test. We assessed children’s baseline heart rate via radial palpation for one minute prior to the start of the test following their participation in five minutes of quiet rest. The child then stepped on and off an 8-inch bench for 3 min, keeping a consistent pace (96 beats per minute) dictated by the beat of a metronome. Immediately following the test, we had the child sit down on the step and immediately measured heart rate via radial artery palpation for one minute. We used the difference between pre- and post-step test heart rates as a proxy for children’s cardiovascular fitness, with lower heart rate (decreased scores) following the step test indicative of greater cardiovascular fitness.

*Cognitive Flexibility*. We used measures of preschool children’s cognitive flexibility in this study. In detail, we employed the Dimensional Change Card Sort (DCCS) Test [58]—an established valid measure for cognitive flexibility in children, with this test providing an index for executive function development in 3–5-year-olds [8,50]. During this test, we instructed preschoolers to accurately place the laminated test cards depicting various dimensions—shapes and colors—into one of two plastic boxes according to rules of increasing complexity. We attached a target picture on each of the two plastic boxes that varied color and shape as test complexity increased. Children were asked to match a series of bivalent test pictures (e.g., red trucks, blue stars) to the target pictures on the plastic boxes; first according to shape and then, after several trials, according to color. We also used “switch” trials during which the child was asked changed the dimension being matched. For instance, after four straight trials matching on shape, we asked the child to match on color and then go back to shape; hence requiring the cognitive flexibility to quickly choose the correct stimulus.

We individually administered this test in a private room after giving a demonstration and allowing children to participate in a practice trial for 1–2 min. Details of the demonstration phase are illustrated in step 2 in the instructions [58]. Generally, children took 5–10 min to complete the test. Scores were based on the number of correct responses in each step the child completed, with higher scores representing greater cognitive flexibility. Additionally, each child was given scores of color and shape game performance was based upon participants who sorted 5 or more out of 6 trials correctly. Scores on the border game were based upon the number correct out of 12. More details of scoring can be found in previous studies [50,58]. The internal consistency of the test was 0.75 in the present study, indicating the test had acceptable reliability in preschool children [50]. Recent studies confirmed that this instrument has been valid in assessing changes of cognitive flexibility among preschool children during a 12-week intervention [8,50].

### 2.4. Intervention

*Exergaming Intervention:* Parent–child dyads randomized into the study were contacted by phone following the completion of baseline measures. We used this phone call to schedule a home visit at a time convenient for the parents and child(ren). During the home visit, we provided children a LeapTV gaming console, all necessary peripherals, and several age-appropriate educational exergames (dance and sports) for use during their time completing the educational exergaming intervention. We set up and demonstrated the use of the LeapTV console while also providing a link to tutorials that parents and their child(ren) could use to guide them through the learning process. Parents were instructed to have their children perform educational exergaming on the LeapTV for 30 min/session, 5 times per week, for 12 weeks, and we developed specific daily gameplay duration goals with the parents to ensure this dosage was achieved. Parents tracked their child(ren)’s achievement of these goals using a pre-distributed calendar upon which stickers were placed as child(ren) met their daily duration goals. We called the parents 2 days after LeapTV installation to ensure that there were no technical issues, and we visited each family once or twice within the first two weeks after installation to encourage continued use of the LeapTV gaming console and discuss any barriers to educational exergaming use. While we initially provided parents LeapTV games classified as ‘easy’ to reduce any difficulty, children with low motor skills had learning to play the LeapTV games, we encouraged parents to visit the lab once monthly to switch their old games with different games to increase child(ren)’s gameplay interest and motivation. As such, preschool children had a variety of PA choices and autonomy, which might promote PA participation, particularly during harsh winters of the Midwestern state in which the study occurred. We discouraged home-based sedentary video game play during the child(ren)’s time in the intervention condition. Rather, we recommended the parents to implement daily exergaming (active screen time) to replace children’s passive screen time (e.g., watching TV, computer, etc.) at home (but not traditional sports and PA). After the 12-week exergaming intervention, we visited the family again and conducted the final survey in a face-to-face fashion.

*Control:* We requested that children maintain their regular PA patterns during their time in the control condition, with exergaming gameplay prohibited. Meanwhile, we also discouraged sedentary video game play at home for preschool children during the intervention period. After the 12-week intervention, the children in the control condition received the gaming console, all necessary peripherals, and exergames (dance and sports) for another 12 weeks as the incentive of participating in this study.

*Intervention Fidelity:* We continuously monitored intervention fidelity during each child’s completion of the educational exergaming intervention on a monthly basis via phone interviews and/or home visits. Briefly, we used a standard phone script for each phone survey/home visit and completed a checklist indicating components of the intervention protocol that were appropriately covered during the observed home-based educational exergaming session. The phone survey included parental surveys about child’s screen time at home related to TV, media, and games; child(ren)’s gaming use and satisfaction. Any intervention component determined to fall outside the predefined minimum implementation requirements was targeted for further staff training and problem solving to address implementation barriers, with intensified efforts continued until the fidelity level reached the targeted goal. Specifically, we met regularly with the research assistants and discussed implementation issues observed within a given child(ren)’s home and develop a goal setting and behavioral tracking program with parents. Intervention fidelity was defined as ≥90% of the intervention components having been covered during the observed home-based educational exergaming session; thus goal setting and behavioral tracking used to increase intervention fidelity sought to achieve this 90% target. Parents received $100 as an incentive for appropriate intervention implementation following the intervention.

### 2.5. Procedures

We obtained University Institutional Review Board approval (number: 1503M65541) and parental consent/child assent prior to study initiation. Interested families contacted us via phone or email after which we screened parents for interest and eligibility with a pre-determined phone script (see the attached phone script; families contacting us via email scheduled a phone interview). If eligible and interested, parents scheduled a baseline assessment for their children within our lab. We confirmed the eligibility of parents and child(ren) once more upon the parents and child(ren) arriving to the lab for baseline assessments—obtaining parental consent/child assent at this time.

We conducted all measurements in a blinded fashion, with the measurement staff blinded to the children’s group allocation. All children underwent identical assessments during the two waves of assessments.

### 2.6. Data Analysis

We analyzed data with SPSS 25.0 (IBM Inc., Armonk, NY, USA). Screening for non-normality and outliers was conducted prior to the main analysis, with missing data imputed using the expectation maximization approach [59]. Descriptive statistics (e.g., mean, standard deviation, etc.) were reported for all outcome variables. A series of two-way (2 groups: exergaming vs. control) analysis of covariance with repeated measures (time: baseline vs. post-intervention) were performed to determine if significant differences occurred on preschool children’s EE, cardiovascular fitness, body mass index and cognitive function, with gender as covariate. The within-subject factor was time (baseline vs. post-intervention) and the between-subject factor was group (i.e., exergaming intervention vs. control). The test of interest was the time × group interaction assessed in the present study. Significance was set at *p* < 0.05 for all analyses, while effect size for each comparison was measured by Partial Eta Square (*η*^2^). In the present study, small, medium, and large effect sizes were designated as 0.01 ≤ 0.06, 0.06 < 0.14, and ≥ 0.14, respectively [60].

## 3. Results

Of the 34 preschool children randomized, 2 children did not complete the study, leading to a final sample size of 32 (94.12% retention). The reason for the missing data was non-compliance with the research protocols. The final sample consisted of 16 boys and 16 girls (*M*_age_ = 4.72 years; *SD* = 0.73), with the racial distribution including 19 Asian Americans, 11 White Americans, and 2 African Americans. To discern if the exergaming intervention group and control group were identical regarding demographic and anthropometric data at the baseline, we conducted independent t-tests for continuous data and Chi-square tests for categorical data. The data suggested that there were no significant differences on any sample characteristics across these two groups at baseline (see Table 1). 

The analysis of covariance with repeated measures yielded significant time x group interaction effects for cognitive flexibility, Wilks’ Lambda = 0.81, *F*(1, 29) = 6.98, *p* = 0.01, *η*^2^ = 0.19 (See Table 2). Specifically, preschool children in the exergaming group demonstrated significantly greater increases in cognitive flexibility as compared to those in the control group over the course of 12 weeks. The effect size was medium for this comparison. Nevertheless, there were no significant differences for time x group changes between the two groups for EE, Wilks’ Lambda = 0.92, *F*(1, 29) = 2.29, *p* = 0.14, *η*^2^ = 0.08; cardiovascular fitness, Wilks’ Lambda = 0.96, *F*(1, 29) = 1.07, *p* = 0.31, *η*^2^ = 0.04; and BMI, Wilks’ Lambda = 0.99, *F*(1, 29) = 0.05, *p* = 0.82, *η*^2^ = 0.01. In other words, preschool children’s EE, cardiovascular fitness and BMI in the exergaming group did not increase significantly more over time relative to children in the control group. The effect sizes for these three comparisons were null to less than medium (*η*^2^ = 0.06).

Notably, no significant interaction effects of time x gender (covariate) were identified indicating that boys and girls did not differ on any outcomes over time. In addition, our data suggested that time effect for EE approached significant level, Wilks’ Lambda = 0.89, *F*(1, 29) = 3.26, *p* = 0.08, *η*^2^ = 0.08, indicating that overall, young children’s daily EE slightly improved from baseline to post-intervention with medium effect size. However, no significant group effect was identified for any outcomes.

## 4. Discussion

Exergaming has been increasingly incorporated into numerous community- and school-based programs due to its potential to promote PA and health in youth and adults [31,32,33,34,35,36,37,61,62,63]. To our knowledge, our study is the first investigation of home-based educational exergaming program on preschool children’s EE, cardiovascular fitness, body mass index, and cognition in the U.S. Our data yielded mixed findings concerning the effects of educational exergaming on these outcomes.

Observations indicated slight higher EE at baseline for preschool children in the educational exergaming condition vs. the control, but there were no significant differences on the increases in EE between the groups over time. While intervention children’s EE increase was not significant post-intervention, our data echo previous studies which suggested exergaming could increase children’s EE [28,34,64]. For instance, Gao and associates indicated that children participating in a school-based exergaming intervention had significantly higher EE than children within a control group [30]. Graf and colleagues also noted children to demonstrate a twofold increase in EE while playing exergaming dance games relative to watching TV [28]. Our study observations extend the previous findings in older children to early childhood. We speculate that increased EE in exergaming may be due to greater perceived enjoyment of educational exergaming in children—possibly resultant from the current generation of children’s interest in video and computer interaction [34,37,38]. In the present study, children in the control group also had non-significant increased EE over time. Further exploratory research is needed.

Children from both groups demonstrated slightly increased body mass index over time during their participation in educational exergaming or usual practice. Importantly, the mean body mass index of children randomized to both conditions was within the healthy range for this age group—suggesting that other developmental factors might have played a role in these observations. Previous studies have examined the effects of exergaming on older children’s body composition. For example, Staiano et al. [65]. implemented a 24-week home-based exergaming in children aged 10–12 years old and found that the intervention improved children’s body mass index z-scores but not dual energy X-ray absorptiometry-assessed body fat percentage. Graves and colleagues, [66] working with 8–10 year old children, revealed no significant differences in body fat percentage between children participating in a 12-week exergaming intervention and control children. By contrast, Maddison et al. [64]. suggested that, by the end of their 24-week exergaming intervention, children (aged 10–14) from the intervention group demonstrated a reduction in body fat percentage. The present study identified different change patterns in body mass index of children from intervention group and control group. The differences between our study’s observation and prior studies may be attributable to the younger population targeted. Indeed, a child’s body composition is impacted by multifaceted factors such as genetics, nutrition, and environmental influences [18,19,20,21]. Hence, we recommend future research consider other confounding factors like eating behavior, length of PA program, and family support while examining the effectiveness of PA programs in early childhood.

Interestingly, no matter the condition a child was randomized to, we identified non-significant increased cardiovascular fitness in preschool children. Yet, intervention children had a slightly yet non-significantly greater increase in fitness vs. control children over time. Researchers indicated short-term exergaming programs could capture and maintain children’s intrinsic motivation and enhance a health-enhancing level of fitness in lab and field settings [37,39,44,62]. The present study offered data-based support for employing exergaming program to improve preschool children’s cardiovascular fitness over 12 weeks at a home setting.

Finally, although both groups improved cognitive flexibility over the course of 12 weeks, the children randomized to receive the intervention had significantly greater improvements than children randomized to the control. Previous empirical evidence suggests that PA could enhance cognitive functioning (e.g., cognitive flexibility, working memory) in children [67,68,69]. Research evidence has also shown that PA can increase students’ concentration and mental cognition, and reduce fidgeting or other self-stimulatory behaviors [70,71]. For example, students often are more attentive and behave better after participation in PA during recess or classroom-based PA [72,73]. Exergaming, as an innovative PA that combines PA and video games, may produce more benefits due to the positive effects of video games on children’s cognitive development [50]. In the past decades, researchers have suggested that video games can yield positive effects on children’s cognition such as working memory, executive functions, and decision making, among others [74,75,76,77,78]. Moreover, researchers recently have observed that exergaming produced immediate gains in cognition in elementary school children [79] and short-term gains in cognition in preschool children [50]. Our study’s observations partially corroborate previous studies and suggest that exergaming may have the potential to enhance young children’s cognitive flexibility.

The strengths of the current study include the application of a novel, developmentally appropriate, and quality educational exergaming program in early childhood and objective assessments of preschool children’s EE, cardiovascular fitness, body mass index, and cognition. However, several limitations should be noted to direct future research. First, the sample size of this study was modest, and we only recruited preschool children from one urban area in the U.S. Given that two children dropped out from the control group, and that the sample size of this group was less than ideal, the findings of the present study may not be generalized to young children from other geographic areas and background. Therefore, a larger and more diverse sample may be recruited in the future. Second, the present study only included LeapTV games as the educational exergaming intervention content. It is possible that young children’s passion and enjoyment may have faded as time went by. It is, therefore, recommended to adopt a large variety of exergaming (e.g., Nickelodeon Fit, Just Dance for Kids) in the future intervention programs. Additionally, the 12-week intervention length may not be ideal. Thus, longer intervention lengths with quality programs among preschool children should be used in future studies. Finally, we checked with parents regarding children’s engagement in exergaming or regular activities through monthly phone check-up, but we did not track preschool children’s passive screen time in this study. Thus, there was no way that we were able to discern the status of children’s passive screen time and/or if these children used active screen time to replace passive screen time. It is imperative to assess this outcome in future home-based studies.

In summary, participation in the home-based exergaming program has potential to improve preschool children’s cognition. No known studies have yet examined the effects of a developmentally appropriate and innovative educational exergaming intervention on preschoolers’ physical health and cognition at the home setting in the U.S. This project is significant because it examined an innovative technology-based intervention that promotes cognitive outcomes in preschoolers. The findings of the current study offer important implications to health professionals working with young children. Specifically, our results suggest that exergaming facilitates preschool children’s cognitive flexibility while not sacrificing other physical outcomes like EE and BMI. Therefore, educational exergaming program may be considered as part of the PA programs at home [80]. Conversely, challenges exist while implementing educational exergaming in the home setting. For example, some young children with low levels of motor skills or fitness might encounter a potentially long and frustrating learning process with some gaming activities. Hence, each game’s Tutorial Mode and/or parental/research staff instructions should be used to guide preschool children through the learning process. Also, possible technical issues with the exergaming systems may occur during the intervention period. Researchers and/or health professionals should work closely with the parents and send personnel on a regular basis to fix any technical problems as early as possible to ensure high intervention fidelity. Taken together, the findings inform the development of innovative PA programs at home as a means to promote optimal development in underserved young children. Health professionals may adopt exergaming at home to help young children development a healthy and active lifestyle with the promote health and cognitive development [81,82,83].

## Figures and Tables

**Table 1:** Characteristics of children.

Variables	Control (*n* = 14)	Intervention (*n* = 18)	*p* Value *
Age, years	4.93/0.83	4.56/0.62	0.15
Gender			0.15
Boys, *n* = 16	5	11	
Girls, *n* = 16	9	7	
Race/ethnicity			0.16
White American, *n* =11	7	4	
Asian American, *n* =19	7	12	
African American, *n* =2	0	2	
Height, cm (M/SD)	111.46/8.21	108.83/6.91	0.33
Weight, kg (M/SD)	18.86/2.90	18.21/2.58	0.50
BMI, kg/m^2^ (M/SD)	15.14/1.24	15.32/1.12	0.67

M = mean; SD = standard deviation. *: Student’s *t*-test for continuous variables and Chi-square test for categorical variables.

**Table 2:** Preschool children’s energy expenditure, cardiovascular fitness, BMI, and cognition across time.

	Intervention Group			Control Group			Overall Sample		
	Baseline	13th Week	Mean Diff	Baseline	13th Week	Mean Diff	Baseline	13th Week	Mean Diff
EE	357.42 /106.36	363.42 /112.33	6.07 /108.95	338.67 /74.25	384.59 /105.5	45.92 /64.27	349.29 /92.80	372.59 /68.08	23.30 /93.07
Fitness	10.22 /6.75	7.56 /4.18	−3.06 /8.33	10.64 /5.58	10.57 /3.72	−0.46 /7.22	10.41 /6.17	8.88 /4.20	−1.53 /7.75
BMI	15.32 /1.12	15.42 /1.35	0.15 /0.65	15.14 /1.24	15.34 /1.38	0.14 /0.64	15.24 /1.15	15.39 /1.34	0.15 /0.65
Cognition	50.89 /7.70	59.39 /7.59	8.76 /5.99	59 /7.65	62.36 /9.19	3.54 /5.62	54.44 /8.59	60.36 /9.19	6.25 /6.20

Note. EE = energy expenditure; Fitness = cardiovascular fitness; BMI = body mass index; M/SD = Mean/Standard Deviation; Mean diff = Mean difference between 13th week and baseline.
